# Effect of music therapy combined with aerobic exercise on sleep quality among breast cancer patients undergoing chemotherapy after a radical mastectomy: a randomized controlled trial

**DOI:** 10.1186/s12905-024-03241-6

**Published:** 2024-07-18

**Authors:** Li Chang, Yan Wang, Jie Zhang, Wenqian Zhao, Xiaomei Li, Lei Yang

**Affiliations:** 1 Shaanxi No. 3 People’s Hospital, Shaanxi Provincial Tumor Hospital, Xi’an, 710061 Shaanxi China; 2 School of Nursing, Health Science Centre, Xi’an Jiaotong University, Xi’an, 710061 Shaanxi China

**Keywords:** Music therapy, Aerobic exercise, Sleep quality, Breast cancer, Radical mastectomy, Randomized controlled trial

## Abstract

**Purpose:**

We aimed to study the effect of music therapy combined with aerobic exercise on the sleep quality of patients undergoing chemotherapy after a radical mastectomy.

**Methods:**

A randomized controlled trial was conducted at the Breast Disease Diagnosis and Treatment Center, Shaanxi Province Tumor Hospital, from July 2017 to June 2019. 110 female breast cancer patients who underwent a radical mastectomy were recruited and randomly allocated into an intervention group or a control group. The intervention group completed music therapy combined with aerobic exercise from the first to the sixth admission to the hospital for chemotherapy, while the control group received only routine nursing care. The sleep quality of these patients was measured using the Pittsburgh Sleep Quality Index (PSQI). A linear mixed model was used to adjust the PSQI of patients after controlling for other confounding factors.

**Results:**

The mean sleep quality score of the breast cancer patients who received chemotherapy after a radical mastectomy (baseline) was 8.86 ± 2.34. The intervention group had a significantly lower mean global PSQI score than the control group from the first test to the third test, with an adjusted mean difference of -1.05 (95%CI: -1.86, -0.24; *P* = 0.01), -2.89 (95%CI: -3.70, -2.08; *P* < 0.001) and − 4.84 (95%CI: -5.65, -4.03; *P* < 0.001), respectively. A change in the global PSQI score from baseline for the intervention group was from 0.55 (95%CI: -0.24, 1.34; *P* = 0.171) at the first test to 2.75 (95%CI: 1.96, 3.53; *P* < 0.001) at the last test, and for the control group was from − 0.51 (95%CI: -1.31, 0.29; *P* = 0.213 at the first test to -2.10 (95%CI: -2.91, -1.30; *P* < 0.001) at the last test.

**Conclusions:**

An intervention of music therapy combined with aerobic exercise can significantly improve the sleep quality of female breast cancer patients undergoing chemotherapy after a radical mastectomy, and this intervention continuously improves many aspects of sleep reactivity.

**Clinical trial registration:**

This study was registered in the Chinese Clinical Trial Registry (ChiCTR2100042975, 02/02/2021).

## Introduction

Breast cancer is the most common cancer in the vast majority (83%) of countries and the leading cause of cancer deaths in more than 100 countries [1]. There were approximately 2.3 million new breast cancer cases worldwide in 2020, accounting for nearly a quarter of female cancer cases, with age standardized incidence and mortality rates of 47.8 per 100,000 and 13.6 per 100,000, respectively [1]. With improvements in the diagnosis of breast cancer and the wide application of effective, comprehensive treatment methods, the survival time of breast cancer patients has been prolonged [2]. However, advances in cancer treatment also cause cancer-related issues, including physical and psychological dysfunction and impacting health-related quality of life [3–5]. For example, adverse reactions to chemotherapy can seriously impact the sleep quality of patients with breast cancer [6]. Therefore, effective strategies should be proposed for the care of breast cancer survivors care to improve their quality of life.

Sleep is the critical biological behavior to maintain the best immunity, endocrine function, and cell metabolism, and it also plays an essential role in regulating hormone levels, emotion, and cognitive behavior [7, 8]. Sleep disorder is usually combined with anxiety, depression, cancer fatigue, pain, and other symptoms, which reduce the quality of life of patients with breast cancer; therefore, having a sleep disorder is a risk factor for the progression, metastasis, and prognosis of breast cancer [7]. In recent years, with the increasing awareness of the importance of the quality of life of cancer patients, improving the sleep quality of patients has become a major focus of cancer research [9].

Music therapy has been widely used as a complementary therapy for cancer patients. It has been shown that music therapy had beneficial effects on anxiety, depression, pain management, fatigue and quality of life of cancer patients [10]. However, not all types of music therapy are equally effective. For instance, music with a slow tempo and harmonious melody tends to be more beneficial for improving sleep quality [11]. A study found that music therapy could reduce pain and anxiety and promote sleep quality of cancer patients receiving chemotherapy [12]. Furthermore, music therapy can interfere with the release of substances such as morphine, relieve the anxiety and depressive mood of the patient, and further play a sedative and hypnotic effect [13]. On the other hand, aerobic exercise has been proven to reduce the physical and psychological effects of cancer and its treatment, improve neuroendocrine function and increase the levels of serotonin and endorphin [14, 15]. These effects can help relieve fatigue, enhance body function, and improve cardiopulmonary function as well as the psychological and mental state, which are important for improving the sleep quality [16, 17]. A recent study showed that an aerobic and resistance exercise intervention effectively improved patient-reported sleep quality in breast cancer survivors [18].

However, research has shown that not all types of exercise and music therapy have the same impact on sleep quality. For example, while aerobic exercise has been widely recognized for its benefits in improving sleep [17, 18], other forms of exercise such as resistance training and stretching have shown mixed results [19]. Additionally, the intensity, duration, and type of aerobic exercise must be carefully tailored to the individual’s condition to achieve the desired sleep improvements [20]. Similarly, the effectiveness of music therapy can vary depending on the type of music used, the duration of the therapy, and individual patient preferences [10, 12, 21]. Studies have found that classical and slow-tempo music tend to be more effective in promoting relaxation and improving sleep quality compared to other genres [11, 22, 23].

Because cancer survivors generally face various cancer-related complications that are influenced by a number of factors, the combination of different therapy approaches is gaining interest [24]. It was found that music therapy combined with other rehabilitation strategies is an effective modality for improving physiological stability, and aerobic exercise combined with music therapy is more effective in improving depression and general discomfort in individuals with fibromyalgia than aerobic exercise along [25]. Increasing evidence showed that both aerobic exercise and music therapy are effective for cancer patients separately. However, many studies employ varied intervention protocols. For example, the duration and frequency of music therapy sessions can vary significantly, a study used 30-minute sessions twice a week​​, while another study employed daily sessions lasting one hour [11, 26]. Similarly, aerobic exercise interventions differ in terms of intensity, type (e.g., walking, cycling, swimming), and session length. Some studies focus on moderate-intensity walking for 30 min, three times a week​, while others use high-intensity cycling sessions for 45 min, five times a week​ [17, 18, 27]. These inconsistencies make it challenging to compare results across studies and draw definitive conclusions about the most effective intervention protocols.

Despite the advantages of music therapy and aerobic exercise mentioned above, the gap of knowledge about the effect of the combined music therapy and aerobic exercise on the sleep quality of breast cancer patients undergoing chemotherapy after a radical mastectomy is far from being understood. Therefore, the objective of the present randomized controlled trial (RCT) was to study the effect of music therapy combined with aerobic exercise on the sleep quality of breast cancer patients after a radical mastectomy. The intervention protocols of our study were designed in accordance with existing guidelines and research findings, and using a structured framework, to ensure consistency and reliability in assessing the combined effects of music therapy and aerobic exercise on sleep quality.

## Materials and methods

### Study design and participants

The present study was a parallel, two-arm, single-center, open-labeled RCT conducted at the Breast Disease Diagnosis and Treatment Center, Shaanxi Provincial Tumor Hospital, from July 2017 to June 2019. The trial protocol was reviewed and approved by the Human Research Ethics Committee of Shaanxi Provincial Tumor Hospital (No.2017 − 148). This study was registered in the Chinese Clinical Trial Registry (ChiCTR2100042975, 02/02/2021).

The target population was female breast cancer patients who received chemotherapy about 10–15 days after a modified radical mastectomy or an extensive radical mastectomy. The chemotherapy regimens included docetaxel, epirubicin, and cyclophosphamide (TEC); dense adriamycin and cyclophosphamide (AC); sequential dense paclitaxel or docetaxel; AC; or docetaxel and cyclophosphamide (TC). The personalized doses of these drugs were calculated based on the patient’s weight and body surface area. All included patients received 6–8 complete treatment cycles, with each cycle consisting of 8 days of treatment followed by 21 days of no treatment.

The patients were recruited in this study by the convenient sampling method if they met the following inclusion criteria: [1] they were willing to join the study voluntarily; [2] aged 18 years or order; [3] they were diagnosed with breast cancer by tumor, lymph node, metastasis (TNM) staging based on the fast pathology slide and paraffin section; [4] they were undergoing chemotherapy after a radical mastectomy. By a detailed interview and assessment, patients with consciousness disorders; mental or neurological disorders; mental, hearing, or cognitive impairments; physical disabilities; or breast cancer with other cancers were excluded. Stage IV breast cancer patients were also excluded as their life expectancy was short. Before enrollment, the patients were given all related information about the study and signed the informed consent forms.

### Randomization and blinding

Following baseline assessment, the patients were randomly allocated into the experimental group for music therapy combined with aerobic exercise and control group for routine nursing care. Based on the hospital identification number of each patient, all the patients were randomly allocated to two groups using a randomized code generated by computer software. The randomization was completed by independent research assistant who was not involved in recruitment, intervention, or data collection. The allocation of participants was conducted by using consecutive numbered, sealed, and opaque envelopes. Due to the intervention nature of this study, the patients and care providers were not blinded to the interventions. Besides, the data collectors and the outcome assessors were also not blinded to the intervention.

### Intervention procedures

#### Music therapy combined with aerobic exercise

The intervention group completed music therapy combined with aerobic exercise from the first admission to the hospital for chemotherapy to the sixth admission to the hospital for chemotherapy and received routine nursing care. The researchers regularly visited the patients in the experimental group during the intervention period and supervised them to ensure that they adhered to the music therapy combined with aerobic exercise procedures. In addition, during the intermittent period of chemotherapy treatment, the patients in the intervention group were followed up by telephone and encouraged to adhere to the intervention procedures. The detailed regimen of the treatment is described below.

The design of music therapy was based on previous studies, and the establishment of music media library was according to the characteristics of Pentameter Therapy Principle and different tonality [28, 29]. The standardized music therapy protocol included: [1] Environment: Patients gathered in a quiet and comfortable environment to relax their minds and bodies in the patient service center [2]. Frequency and duration: Music therapy was administered two times a day, 30 min each time, from the first admission to the end of the sixth cycle of chemotherapy [3]. Music selection: Patients could choose music according to their preference from a library consisting of 334 instrumental music pieces, including Chinese classical folk music, world-famous music, a Music Therapy Association-recommended nature music series CD, and relaxing music. Specific tracks included “Yao Nationality Dance,” “Two Springs Reflect the Moon,” “Blue Danube River,” “Spring,” “Clouds Chasing the Moon,” and “Destiny Symphony.” Songs and operas were excluded to avoid the interference effect of lyrics [4]. Equipment: Patients used headphones for the treatment, with the volume set at 30–50 dB [5]. Monitoring: Researchers ensured that patients adhered to the protocol during each session and provided regular follow-up and encouragement during non-treatment intervals.

The design of aerobic exercise was based on the American College of Sports Medicine roundtable on exercise guidelines for cancer survivors and a previous study [30, 31]. The standardized aerobic exercise protocol included: [1] Warm-up: Patients performed aerobic exercise for 15–20 min by exercising the upper extremity joints and the whole body, including head rotation, single shoulder exercises, arm exercises, forward bends, hip exercises, jump around a step, and step and swing arms [2]. Exercise routine: The whole set of movements was repeated 2–3 times, for an additional 20–25 min. The practical exercise intensity of the whole exercise session was controlled within the range of the target heart rate of the patients, with the target heart rate = (220 – age – resting heart rate) × (45–60%) + resting heart rate [3]. Cool-down: Cool-down activities were performed for another 10 min, including jogging in place, upper limb swing, and relaxation [4]. Frequency: Group training aerobic exercise was conducted every Monday, Wednesday, and Friday afternoon, until the end of the sixth cycle of chemotherapy. Weekly supplementary training was provided for those who could not exercise at the specified time for special reasons [5]. Monitoring: Intervention team members visited the patients regularly during the hospital stay, helped the patients adhere to the intervention, and conducted regular telephone follow-ups during the nontreatment interval to encourage the patients to continue the intervention.

### Routine nursing care

The control group patients received the same perioperative and chemotherapy routine nursing care. Before chemotherapy, the responsible nurses explained post-radical mastectomy and chemotherapy to the patients. Then, in each chemotherapy cycle, the patient was instructed to perform rehabilitation exercises, including fist-clenching, wrist wrapping, elbow flexion, shoulder, and upper limb joint activities, 3 to 5 times a day for 5 to 10 min each time, until the end of the sixth chemotherapy cycle. However, they did not receive the intervention of music therapy combined with aerobic exercise.

### Data collection

In this study, the social and demographic data as well as the disease-related conditions of the patients were collected by a general questionnaire. The questionnaire was designed by the Breast Disease Diagnosis and Treatment Center of Shaanxi Province Tumor Hospital and was completed under the guidance of relevant experts.

The sleep quality of the patients was evaluated by the Chinese version of the Pittsburgh Sleep Quality Index (PSQI) [32]. The PSQI was designed by Pittsburgh psychiatrist Buysse organically to evaluate the sleep quality and quantity simultaneously in one month [33]. The PSQI includes 19 self-rated and five peer-rated items, of which the 19th self-rated item and all five peer-rated items were not used for the score. The remaining 18 self-rated items were divided into seven components: subjective sleep quality, fall-asleep time, sleep duration, sleep efficiency, sleep disturbance, use of sleep medication, and daytime function, each of which was scored as 0–3. Based on the sum of the seven components with a global score range of 0–21, a higher global PSQI score indicated a worse sleep quality [33]. Therefore, this study set a global score of > 7 as the boundary value of overall sleep quality problems among participants [32]. Besides, we used a cut-off score of ≥ 2 to represent having sleep problems in each component.

Previous psychometric studies of the Chinses version of the PSQI have confirmed that it is a reliable and valid instrument for assessing subjective sleep quality in different populations, with a Cronbach alpha of 0.71 to 0.84 [32, 34, 35]. Therefore, in the present study, the Chinese version of the PSQI was assessed at four testing times to evaluate the sleep quality of the patients in the two groups: at the end of the 10th day after the radical mastectomy (baseline), after the first cycle of chemotherapy (first test), after the third cycle of chemotherapy (second test), and after the sixth cycle of chemotherapy (third test).

### Outcomes

The primary outcome was the change in the global PSQI score from baseline. The secondary outcomes were the changes in each component of the PSQI score (i.e., sleep quality, fall-asleep time, sleep duration, sleep efficiency, sleep disturbance, use of sleep medication, and daytime function) from baseline.

### Sample size

The sample size for this study was calculated using the G-power software (version 3.1.9.7). The calculation was based on the results from a pretest which observed a moderate effect size of 0.6. To achieve a power of 0.8 and a significance level of 0.05, the sample size was estimated to be 90 participants (45 for each group). Considering a 20-25% drop-out rate, we defined the target sample size as 110.

### Statistical analysis

A linear mixed model accounting for repeated measurements was used to assess the effect of the intervention of music therapy combined with aerobic exercise on the sleep quality of the patients after controlling for all of the explanatory variables and the interaction between the intervention variable and the time variable.

In this study, the explanatory variables included the following: (i) age (20–39 years old, 40–59 years old, or ≥ 60 years old); (ii) marital status (unmarried, married, divorced, or widowed), (iii) education level (primary school or lower, junior school, high school, or college and above), (iv) household income per month (≤ 2000 RMB, 2000–5000 RMB, or ≥ 5000 RMB), (v) medical insurance (none, rural cooperation medical service, or other insurance), (vi) work status (unemployed, employed, or retired), (vii) clinical TNM stage (I stage, II stage, or III stage), (viii) chemotherapy regimen (AC sequential paclitaxel or docetaxel, TEC, or TC), and (ix) side effects of chemotherapy (mild, moderate, or severe). The intervention variable (intervention group and control group) and time variable (baseline, after the first, after the third, and after the sixth chemotherapy) were involved in this study.

Data were entered into Epidata, version 3.0 (Centers for Disease Control and Prevention, Atlanta, GA, USA), and all statistical analyses were performed using Statistics Analysis System software, version 9.4 (Cary, NC, USA). Categorical variables are expressed as the count and percentage/proportion. Continuous variables are expressed as the mean with standard deviation. The comparison of categorical variables was performed by Pearson’s chi-squared test or Fischer’s exact test. The statistical significance was achieved from statistical tests when *P* < 0.05.

## Results

### Study participants

**Fig. 1:**
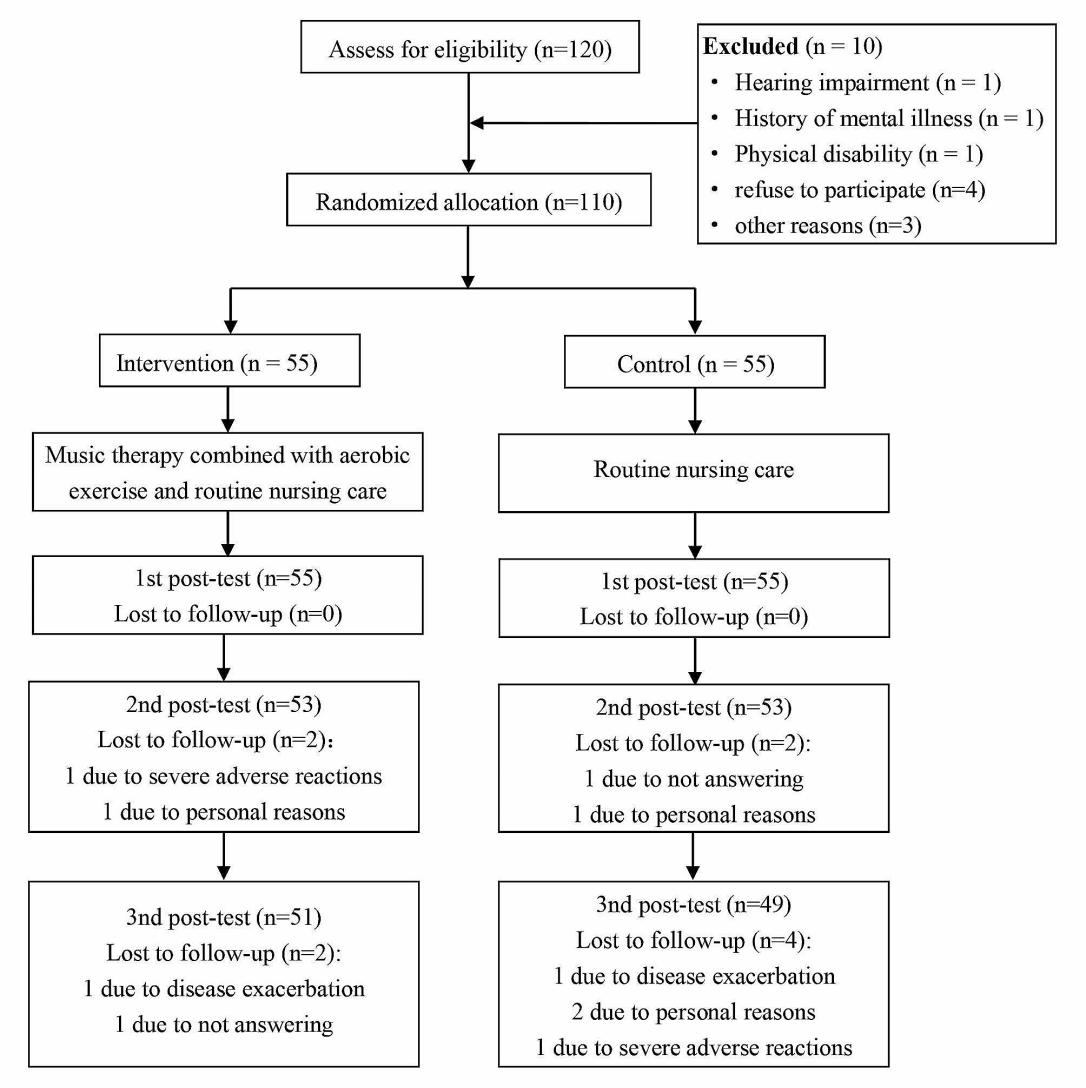
Flow chart of the study

We recruited the patients admitted to our hospital from July 2017 to June 2019, and the participant flow is described in Fig. 1. First, A total of110 patients were recruited according to the eligibility criteria and all of them agreed to participate and signed the consent forms. During the following treatments, four patients in the intervention group and six patients in the control group were lost to follow-up. Reasons for dropping out were severe adverse reactions during chemotherapy (*n* = 2), disease exacerbation (*n* = 2), not answering (*n* = 2), and personal reasons (*n* = 4). Finally, 51 patients in the intervention group and 49 patients in the control group completed the study.

A total of 100 eligible participants were involved in data analysis. Their average age was 44.73 ± 11.697 years old (range: 25–71 years old), and 51% of them were 40–59 years old. Married participants accounted for 86% of the participants. In addition, the income of roughly 21% of the households was more than 5000 RMB each month. Medical insurance, including rural cooperative medical service as well as urban, social, and commercial insurance, covered 99% of patients. Moreover, approximately 63% of the patients had permanent jobs, 66% of the patients had stage II breast cancer, and 88% of the patients experienced moderate side effects during chemotherapy (Table 1).

**Table 1:** Baseline characteristics of patients with breast cancer

Baseline characteristics	Total number (*n* = 100)		Intervention group (*n* = 51)		Control group (*n* = 49)		χ^2^	*P*
	*n*	%	*n*	%	*n*	%		
**Age(year)**							1.410	0.494
20–39	33	33.0	18	35.3	15	30.6		
40–59	51	51.0	27	52.9	24	49.0		
60-	16	16.0	6	11.8	10	20.4		
**Marriage**							1.207	0.751
Unmarried	5	5.0	3	5.9	2	4.1		
Married	86	86.0	42	82.4	44	89.8		
Divorced	6	6.0	4	7.8	2	4.1		
Widowhood	3	3.0	2	3.9	1	2.0		
**Education level**							3.597	0.308
Primary school or lower	19	19.0	8	15.7	11	22.4		
Junior school	20	20.0	13	25.5	7	14.3		
High school	32	32.0	18	35.3	14	28.6		
College and above	29	29.0	12	23.5	17	34.7		
**Household income per month**							0.713	0.700
≤ 2000	26	26.0	14	27.5	12	24.5		
2000–5000	53	53.0	25	49.0	28	57.1		
≥ 5000	21	21.0	12	23.5	9	18.4		
**Medical insurance**							1.827	0.401
No	1	1.0	0	0	1	2.0		
Rural cooperation medical service	54	54.0	30	58.8	24	49.0		
Other insurance	45	45.0	21	41.2	24	49.0		
**Profession**							0.428	0.807
Unemployed	32	32.0	15	29.4	17	34.7		
Working at the job	63	63.0	33	64.7	30	61.2		
Retirement	5	5.0	3	5.9	2	4.1		
**Clinical TNM stages**							0.675	0.714
I stage	8	8.0	5	9.8	3	6.1		
II stage	66	66.0	32	62.7	34	69.4		
III stage	26	26	14	27.5	12	24.5		
**Side effects of chemotherapy**							0.450	0.798
Mild	9	9.0	4	7.8	5	10.2		
Moderate	88	88.0	45	88.2	43	87.8		
Severe	3	3.0	2	3.9	1	2.0		
**Chemotherapy regimens**							0.361	0.835
AC sequential Paclitaxel or Docetaxel	24	24.0	13	25.5	11	22.4		
TEC or TC	50	50.0	26	51.0	24	49.0		
Other	26	26.0	12	23.5	14	28.6		

According to the analysis, there were no significant differences between the intervention group and the control group in terms of patient age, marital status, education level, household income, employment status, medical insurance, cancer clinical TNM stage, course of chemotherapy treatment, or severity of side effects due to chemotherapy (*P* > 0.05) (Table 1).

### Outcomes

The mean sleep quality score of the 100 patients with breast cancer was 8.86 ± 2.34 on the 10th day after their radical mastectomy. According to our records, approximately 76.0% of the patients suffered from sleep problems (a total PSQI score of > 7) on the 10th day after their radical mastectomy. According to the results of this study, 56% of the patients had a poor subjective sleep quality, 42.0% of the patients had trouble falling asleep, and 34% of the patients had sleep disturbance (e.g., easy to get up, get up early, frequently to go the toilet at night). In addition, about 34% of the patients slept < 6 h per night and had daytime dysfunction such as a shortage of energy and fatigue (Table 2).

**Table 2:** The score of pittsburgh sleep quality index among patients (*n* = 100)

	Full score	Min	Max	Score ≥ 2 (%)	M *±* SD
Global score	23	3	18		8.86 ± 2.34
Subjective sleep quality	3	0	3	56.0	1.71 ± 0.74
Fall-asleep time	3	0	3	42.0	1.49 ± 0.66
Sleep duration	3	0	3	34.0	1.34 ± 0.77
Sleep efficiency	3	0	3	38.0	1.43 ± 0.62
Sleep disturbance	3	0	3	34.0	1.42 ± 0.73
Use of sleep medication	3	0	1	0.00	0.02 ± 0.14
Daytime function	3	0	3	40.0	1.45 ± 0.72

A linear mixed model was used to explore the effect of the intervention of music therapy combined with aerobic exercise on sleep quality after adjusting for age, marital status, education level, employment status, household income, medical insurance, cancer clinical TNM stage, and severity of side effects due to chemotherapy. Table 3 presents the findings of the global PSQI score and each component score between two groups at all timepoints. At baseline, the intervention and control groups had similar mean global PSQI score and mean each component score. From the first test to the third test, the intervention group had a significantly lower mean global PSQI score than the control group, the adjusted mean differences was − 1.05 (95%CI: -1.86, -0.24; *P* = 0.01), -2.89 (95%CI: -3.70, -2.08; *P* < 0.001), -4.84 (95%CI: -5.65, -4.03; *P* < 0.001), respectively. Except for the use of sleep medication, the mean score of other components in intervention group was significantly lower than that in the control group at the third test (*P* < 0.001).

**Table 3:** Summary results from mixed model analysis of difference in sleep quality score between two groups at all time points

	Intervention (*n* = 51) Mean ± SD	Control (*n* = 49) Mean ± SD	Mixed model analysis (unadjusted)		Mixed model analysis (adjusted) *	
			Difference (95% CI)	*P* value	Difference (95% CI)	*P* value
**Global PSQI score**						
Baseline	8.82 ± 2.09	8.90 ± 2.60	-0.07 (-0.88, 0.73)	0.856	0.01 (-0.80, 0.82)	0.988
1st post-test	8.27 ± 1.93	9.41 ± 2.00	-1.13 (-1.94, -0.33)	0.006	-1.05 (-1.86, -0.24)	0.011
2nd post-test	7.24 ± 2.02	10.20 ± 1.93	-2.97 (-3.77, -2.16)	< 0.001	-2.89 (-3.70, -2.08)	< 0.001
3rd post-test	6.08 ± 1.61	11.00 ± 2.10	-4.92 (-5.73, -4.12)	< 0.001	-4.84 (-5.65, -4.03)	< 0.001
**Subjective sleep quality**						
Baseline	1.73 ± 0.70	1.69 ± 0.80	0.03 (-0.26, 0.32)	0.829	0.08 (-0.21, 0.38)	0.584
1st post-test	1.57 ± 0.64	1.71 ± 0.71	-0.15 (-0.43, 0.14)	0.321	-0.10 (-0.39, 0.20)	0.526
2nd post-test	1.43 ± 0.78	1.80 ± 0.76	-0.36 (-0.65, -0.08)	0.013	-0.31 (-0.61, -0.02)	0.037
3rd post-test	1.31 ± 0.47	1.92 ± 0.93	-0.60 (-0.89, -0.32)	< 0.001	-0.55 (-0.85, -0.26)	< 0.001
**Fall-asleep time**						
Baseline	1.43 ± 0.61	1.55 ± 0.71	-0.12 (-0.40, 0.16)	0.408	-0.15 (-0.43, 0.13)	0.305
1st post-test	1.31 ± 0.68	1.63 ± 0.70	-0.32 (-0.60, -0.04)	0.028	-0.35 (-0.63, -0.06)	0.016
2nd post-test	1.20 ± 0.89	1.76 ± 0.75	-0.56 (-0.84, -0.28)	0.0001	-0.59 (-0.87, -0.30)	< 0.001
3rd post-test	1.00 ± 0.60	1.96 ± 0.79	-0.96 (-1.24, -0.68)	< 0.001	-0.99 (-1.27, -0.70)	< 0.001
**Sleep duration**						
Baseline	1.33 ± 0.62	1.35 ± 0.90	-0.01 (-0.31, 0.28)	0.928	0.01 (-0.29, 0.31)	0.947
1st post-test	1.16 ± 0.86	1.39 ± 0.84	-0.23 (-0.53, 0.06)	0.124	-0.21 (-0.50, 0.09)	0.169
2nd post-test	1.00 ± 0.72	1.53 ± 0.62	-0.53 (-0.82, -0.24)	< 0.001	-0.51 (-0.80, -0.21)	< 0.001
3rd post-test	0.84 ± 0.58	1.61 ± 0.79	-0.77 (-1.06, -0.47)	< 0.001	-0.75 (-1.04, -0.45)	< 0.001
**Sleep efficiency**						
Baseline	1.41 ± 0.64	1.45 ± 0.61	-0.04 (-0.30, 0.23)	0.783	-0.02 (-0.29, 0.25)	0.877
1st post-test	1.29 ± 0.73	1.57 ± 0.65	-0.28 (-0.54, -0.01)	0.041	-0.26 (-0.53, 0.01)	0.060
2nd post-test	1.08 ± 0.66	1.76 ± 0.72	-0.68 (-0.94, -0.41)	< 0.001	-0.66 (-0.93, -0.39)	< 0.001
3rd post-test	0.88 ± 0.43	1.86 ± 0.89	-0.97 (-1.24, -0.71)	< 0.001	-0.96 (-1.23, -0.69)	< 0.001
**Sleep disturbance**						
Baseline	1.41 ± 0.64	1.43 ± 0.82	-0.02 (-0.33, 0.30)	0.916	0.02 (-0.30, 0.34)	0.901
1st post-test	1.61 ± 0.96	1.59 ± 0.73	0.02 (-0.30, 0.33)	0.920	0.05 (-0.26, 0.37)	0.743
2nd post-test	1.43 ± 0.92	1.67 ± 0.80	-0.24 (-0.55, 0.07)	0.128	-0.21 (-0.52, 0.11)	0.203
3rd post-test	1.16 ± 0.58	1.80 ± 0.82	-0.64 (-0.95, -0.33)	< 0.001	-0.60 (-0.92, -0.29)	< 0.001
**Use of sleep medication**						
Baseline	0.04 ± 0.20	0	0.04 (-0.01, 0.09)	0.109	0.05 (0, 0.09)	0.068
1st post-test	0.02 ± 0.14	0.02 ± 0.14	0 (-0.05, 0.05)	0.974	0.01 (-0.04, 0.05)	0.825
2nd post-test	0	0.02 ± 0.14	-0.02 (-0.07, 0.03)	0.404	-0.01 (-0.06, 0.03)	0.572
3rd post-test	0	0.02 ± 0.14	-0.02 (-0.07, 0.03)	0.404	-0.01 (-0.06, 0.03)	0.572
**Daytime function**						
Baseline	1.47 ± 0.70	1.43 ± 0 0.74	0.04 (-0.27, 0.35)	0.789	0.02 (-0.29, 0.33)	0.913
1st post-test	1.31 ± 0.99	1.49 ± 0.71	-0.18 (-0.48, 0.13)	0.263	-0.20 (-0.51, 0.11)	0.201
2nd post-test	1.10 ± 0.81	1.67 ± 0.82	-0.58 (-0.88, -0.27)	< 0.001	-0.60 (-0.91, -0.29)	< 0.001
3rd post-test	0.88 ± 0.68	1.84 ± 0.77	-0.95 (-1.26, -0.65)	< 0.001	-0.98 (-1.29, -0.67)	< 0.001

* Mixed model adjusted for covariates of age, marriage, household income, medical insurance, profession, clinical TNM stages, course of chemotherapy treatment and side effects of chemotherapy

Table 4 presents the results of the intragroup comparisons. For the primary outcome, a nonsignificant improvement was observed in the intervention group at the first test. However, the global PSQI score from the first test to the third test gradually decreased in the intervention group, with an adjusted change from 0.55 (95%CI: -0.24, 1.34; *P* = 0.171) to 2.75 (95%CI: 1.96, 3.53; *P* < 0.001). On the contrary, in the control group from the first test to the third test, the global PSQI score significantly increased, with an adjusted change from − 0.51 (95%CI: -1.31, 0.29; *P* = 0.213) to -2.10 (95%CI: -2.91, -1.30; *P* < 0.001). With prolongation of the chemotherapy cycles, the global PSQI score, and the sleep quality factor score of the intervention group showed a downward trend, while these scores of the control group showed an upward trend.

**Table 4:** Summary results from mixed model analysis of change in sleep quality score from baseline in intervention and control groups

	Mixed model analysis of change between baseline and by visit (Unadjusted)						Mixed model analysis of change between baseline and by visit (adjusted)*					
	1st post-test (95% CI)	*P* value	2nd post-test (95% CI)	*P* value	3rd post-test (95% CI)	*P* value	1st post-test (95% CI)	*P* value	2nd post-test (95% CI)	*P* value	3rd post-test (95% CI)	*P* value
**Intervention group (** ***N*** ** = 51)**												
Global score	0.55 (-0.25, 1.35)	0.176	1.59 (0.79, 2.39)	< 0.001	2.75 (1.95, 3.54)	< 0.001	0.55 (-0.24, 1.34)	0.171	1.59 (0.80, 2.38)	< 0.001	2.75 (1.96, 3.53)	< 0.001
Subjective sleep quality	0.16 (-0.13, 0.44)	0.280	0.29 (0.01, 0.58)	0.043	0.41 (0.13, 0.70)	0.005	0.16 (-0.1, 0.44)	0.282	0.29 (0.01, 0.58)	0.044	0.41 (0.13, 0.70)	0.005
Fall-asleep time	0.12 (-0.16, 0.40)	0.411	0.24 (-0.05, 0.52)	0.100	0.43 (0.15, 0.71)	0.003	0.12 (-0.16, 0.39)	0.399	0.235 (-0.04, 0.51)	0.092	0.431 (0.16, 0.71)	0.002
Sleep duration	0.18 (-0.11, 0.47)	0.234	0.33 (0.04, 0.62)	0.025	0.49 (0.20, 0.78)	0.001	0.18 (-0.11, 0.46)	0.228	0.33 (0.05, 0.62)	0.023	0.49 (0.20, 0.78)	< 0.001
Sleep efficiency	0.12 (-0.15, 0.38)	0.380	0.33 (0.07, 0.60)	0.013	0.53 (0.27, 0.79)	< 0.001	0.12 (-0.15, 0.38)	0.383	0.33 (0.07, 0.60)	0.014	0.53 (0.26, 0.79)	< 0.001
Sleep disturbance	-0.20 (-0.50, 0.11)	0.212	-0.02 (-0.33, 0.29)	0.901	0.26 (-0.05, 0.56)	0.105	-0.20 (-0.50, 0.11)	0.211	-0.02 (-0.33, 0.29)	0.900	0.25 (-0.05, 0.56)	0.104
Use of sleep medication	0.02 (-0.03, 0.07)	0.418	0.04 (-0.01, 0.09)	0.106	0.042 (-0.01, 0.09)	0.106	0.02 (-0.03, 0.07)	0.418	0.04 (-0.01, 0.09)	0.106	0.04 (-0.01, 0.09)	0.106
Daytime function	0.16 (-0.15, 0.46)	0.313	0.37 (0.07, 0.68)	0.017	0.59 (0.28, 0.89)	< 0.001	0.16 (-0.14, 0.46)	0.305	0.37 (0.07, 0.67)	0.015	0.59 (0.29, 0.89)	< 0.001
**Control group (** ***N*** ** = 49)**												
Global PSQI score	-0.51 (-1.32, 0.30)	0.218	-1.31(-2.12, -0.49)	0.002	-2.10 (-2.92, -1.29)	< 0.001	-0.51 (-1.31, 0.29)	0.213	-1.31 (-2.11, -0.50)	0.002	-2.10 (-2.91, -1.30)	< 0.001
Subjective sleep quality	-0.02 (-0.31, 0.27)	0.890	-0.10 (-0.40, 0.19)	0.491	-0.22 (-0.51, 0.07)	0.130	-0.02 (-0.31, 0.27)	0.891	-0.10 (-0.39, 0.19)	0.493	-0.22 (-0.52, 0.07)	0.132
Fall-asleep time	-0.08 (-0.37, 0.20)	0.576	-0.20 (-0.49, 0.08)	0.162	-0.41 (-0.69, -0.12)	0.005	-0.08 (-0.36, 0.20)	0.567	-0.20 (-0.49, 0.08)	0.152	-0.41 (-0.69, -0.13)	0.004
Sleep duration	-0.04 (-0.34, 0.26)	0.787	-0.18 (-0.48, 0.11)	0.225	-0.27 (-0.56, 0.03)	0.080	-0.04 (-0.33, 0.25)	0.785	-0.18 (-0.48, 0.11)	0.219	-0.27 (-0.56, 0.03)	0.076
Sleep efficiency	-0.12 (-0.39, 0.15)	0.371	-0.31 (-0.57, -0.04)	0.026	-0.41 (-0.68, -0.14)	0.003	-0.12 (-0.39, 0.15)	0.374	-0.31 (-0.58, -0.04)	0.027	-0.41 (-0.68, -0.14)	0.003
Sleep disturbance	-0.163 (-0.48, 0.15)	0.309	-0.245(-0.56, 0.07)	0.127	-0.37 (-0.68, -0.05)	0.022	-0.16 (-0.48, 0.15)	0.307	-0.24 (-0.56, 0.07)	0.126	-0.37 (-0.68, -0.05)	0.022
Use of sleep medication	-0.02 (-0.07, 0.03)	0.408	-0.02 (-0.07, 0.03)	0.408	-0.02 (-0.07, 0.03)	0.408	-0.02 (-0.07, 0.03)	0.409	-0.02 (-0.07, 0.03)	0.409	-0.02 (-0.07, 0.03)	0.409
Daytime function	-0.06 (-0.37, 0.25)	0.699	-0.24 (-0.56, 0.07)	0.123	-0.41(-0.72, -0.10)	0.010	-0.06 (-0.37, 0.24)	0.694	-0.24 (-0.55, 0.06)	0.117	-0.41 (-0.71, -0.10)	0.009

* Mixed model adjusted for covariates of age, marriage, household income, medical insurance, profession, clinical TNM stages, course of chemotherapy treatment and side effects of chemotherapy

Significant improvements from baseline for each component (i.e., sleep quality, fall-asleep time, sleep duration, sleep efficiency, sleep disturbance, and daytime function) of the PSQI for sleep quality were observed throughout the intervention period in both the intervention and control groups from the first test to the third test. A similar trend to that observed for the global PSQI score persisted for these secondary outcomes in the two groups.

## Discussion

The present RCT with 100 breast cancer patients during chemotherapy yielded significant positive effects of music therapy combined with aerobic exercise on subjective sleep quality, fall-asleep time, sleep duration, sleep efficiency, sleep disturbance, and daytime function. The benefit was observed for interventions performed throughout the chemotherapy cycle. These results suggest that the feasible, practical, and nonpharmacological method of music therapy combined with aerobic exercise can significantly improve the sleep quality of breast cancer patients undergoing chemotherapy after a radical mastectomy.

A radical mastectomy of female breast cancer patients makes their breasts incomplete, changes female characteristics, and influences their family roles. Chemotherapy is a main treatment option for breast cancer after radical mastectomy. Along with the side effects of chemotherapy and a significant economic burden, patients often suffer from psychological, physiological, and social disorders. Therefore, negative emotions such as anxiety and depression often accompany them and decrease their sleep quality [36]. In this study, the mean total PSQI score of the participants was 8.86 ± 2.34, which is higher than the general population in China (3.88 ± 2.52) [32] and the level of breast cancer patients reported by Fortner et al. (6.8 ± 4.0) [36, 37]. Sleep disorders are one of the most common symptoms of patients after a radical operation for breast cancer. Besides, the immunity function can be reduced due to severe sleep disorders, which are often associated with anxiety, depression, cancer fatigue, and pain [7, 38, 39]. Therefore, sleep disorders seriously affect the clinical outcomes, physical and mental health, and the quality of life of patients. Our study showed that 76% of the patients suffered from sleep problems, suggesting that they are common in breast cancer patients undergoing chemotherapy after a radical mastectomy. These results were similar to previous studies on the incidence of sleep disorders in patients with breast cancer [40, 41].

The main finding of the present study was that PSQI score of breast cancer patients during chemotherapy was significantly reduced by music therapy combined with aerobic exercise when compared with the ones without the intervention. In addition, music therapy combined with aerobic exercise significantly reduced the global PSQI score and all sleep quality indicators in the intervention group throughout the intervention period from baseline to third test. However, an apparent increase of the PSQI score was observed in the control group from baseline to the subsequent tests. These results suggest that chemotherapy is associated with an increase in sleep problems in breast cancer patients, and routing nursing care measures are not effective for improving sleep quality. This is in line with some previous studies that have reported a deleterious effect of chemotherapy on sleep in breast cancer patients [42, 43].

Music therapy is a type of non-invasive, natural therapy [44]. Through beautiful and harmonious music, patients can have emotional and psychological relief and achieve empathy, suggestion, induction, and other psychologic effects [45]. Music therapy enables people to experience music, eliminate psychological obstacles, restore or improve mental and physical health, and improve the quality of life [46]. It has also been suggested as a potent cost-effective intervention for sleep improvement [11]. Systematic reviews have shown that music therapy can relieve mild-to-moderate pain and effectively alleviate the poor sleep quality caused by negative emotions such as depression, fear, and anxiety in cancer patients [23, 47]. Our previous studies showed that music therapy had both short- and long-term positive effects on alleviating pain and was benefit for improving anxiety in breast cancer patients following radical mastectomy [28, 29]. This study further provided evidence of effectiveness of music therapy on sleep quality in breast cancer patients.

Aerobic exercise is defined as any form of physical activity that produces an increased heart rate and respiratory volume, and that has produced a variety of positive impacts in people with different health conditions. It has been confirmed that aerobic exercise is one of the most effective ways to fight cancer and prevent cancer [48]. Aerobic exercise also has been proven to be one of the primary interventions to improve the overall quality of life, reduce patient mortality, help patients to recover, relieve psychological stress, reduce cancer-related fatigue, and improve the social support status of patients with breast cancer [49, 50]. Besides, aerobic exercise has been reported to have a positive impact on promoting sleep outcomes in cancer patients with poor sleep quality, and the benefits are sustained 3–6 months after the intervention [17]. However, the effects of physical activity on sleep quality have been inconsistent across different studies. A study found that physical activity interventions could improve sleep quality in breast cancer survivors, but there were no significant differences in sleep duration, sleep latency, and habitual sleep efficiency between the intervention and control groups [51]​​. In contrast, Mitchell et al. found no reciprocal associations between daily physical activity and nighttime sleep, suggesting that increased physical activity does not necessarily translate to improved sleep quality [52]​. The variability in findings regarding physical activity may be attributed to differences in the types of exercises, intensity, and duration of interventions, as well as the methodologies used to measure sleep outcomes. Studies using self-reported sleep quality assessments may yield different results compared to those using objective measures like actigraphy​.

A growing number of studies have shown that integrative therapy approaches may improve the symptoms and side effects associated with breast cancer and its treatment [53]. Although the implementation of music therapy combined with aerobic exercise among breast cancer patients has not yet been investigated, the use of combination of music therapy and other treatments in other population has shown beneficial effects. A randomized trial showed that mindfulness-based stress reduction combined with music therapy effectively reduced pain and anxiety scores and improved the sleep quality in patients with osteosarcoma [54]. The inconsistencies in research findings underscore the need for standardized intervention protocols. Our study addresses these gaps by employing a consistent and well-defined protocol for both music therapy and aerobic exercise. Our findings showed a significant improvement of sleep quality in breast cancer patient after the combined intervention of music therapy and aerobic exercise. The PSQI score in the intervention group showed a decreasing trend with an increase in the number of chemotherapy cycles and interventions. However, it is important to note that the success of exercise and music therapy interventions can be influenced by various factors, such as the type of exercise, its intensity, and the genre of music, the patient’s music preferences. Future studies should aim to identify the most effective combinations of these factors to optimize therapeutic outcomes.

To our knowledge, this study first confirmed the effects of music therapy combined with aerobic exercise on sleep quality in breast cancer patients undergoing chemotherapy after a radical mastectomy. The strength of the current study was that we rigorously followed the RCT design procedure. Thus, the cause-effect relationship was able to be confirmed. However, although considerable efforts had been made to perfect our study, this study still had some limitations that must be addressed. First, the patient-reported data could have been influenced by many potential factors, such as defensiveness, misrepresentation, personal emotions, and attitudes. Second, no biomarkers or physiological measurements were collected or analyzed and should be further studied in the future. Third, the synergic effects of music therapy and aerobic exercise have not been assessed. Next, we did not consider other critical health-related endpoints such as quality of life during the study design, which we will study in the future. Finally, because this was a one-center study, the present findings are likely to have a low degree of generalizability to other areas in China.

In conclusion, music therapy combined with aerobic exercise is effective in improving the sleep quality of breast cancer patients undergoing chemotherapy after a radical mastectomy. Our study contributes to this body of knowledge by offering a rigorous examination of combined music therapy and aerobic exercise interventions, paving the way for more effective and tailored treatment strategies for cancer patients. Undoubtedly, further research and more music therapy experience combined with aerobic exercise are still needed. Furthermore, in clinical nursing practice, the patient’s music preference, stress tolerance, and degree of upper limb activity of the affected side should be comprehensively evaluated.

## Data Availability

The datasets used and/or analyzed during this study are available from the first author (changli6213531@163.com) on reasonable request.
